# Reliability and validity of pelvic floor muscle strength assessment using the MizCure perineometer

**DOI:** 10.1186/s12905-020-01127-x

**Published:** 2020-11-19

**Authors:** Yui Abe-Takahashi, Takeya Kitta, Mifuka Ouchi, Minori Okayauchi, Hiroki Chiba, Madoka Higuchi, Mio Togo, Nobuo Shinohara

**Affiliations:** 1 Department of Renal and Genitourinary Surgery, Graduate School of Medicine, Hokkaido University, Kita 15 Nishi 7; Kita-ku, Sapporo, Hokkaido 060-8638 Japan; 2 Department of Physical Therapy, Faculty of Health Sciences, Hokkaido University of Science, Sapporo, Japan; 3 Department of Physical Therapy, School of Rehabilitation Sciences, Health Sciences, University of Hokkaido, Tobetsu, Japan; 4 Department of Rehabilitation, Sapporo Maruyama Orthopedic Hospital, Sapporo, Japan

**Keywords:** MizCure, Vaginal pressure, Reliability, Validity, Pelvic floor muscles, Perineometer

## Abstract

**Background:**

The purpose of this study was to clarify the reliability and validity of pelvic floor muscle (PFM) strength assessment using the MizCure perineometer in healthy women.

**Methods:**

Twenty healthy women (age 20–45 years) participated in this study. The vaginal pressure measured using the MizCure and validated Peritron perineometers were repeated during PFM contraction in the supine and standing positions. All women were evaluated twice by examiners 1 and 2. Following the measurements in the first session (Test 1), they were repeated after an interval of between 2 and 6 weeks (Test 2). Within- and between-session intra- and inter-rater reliabilities in vaginal pressure were analyzed using intraclass correlation coefficients (ICC) (1, 1) and (2, 1), respectively. Validity was assessed by Pearson’s product-moment correlation coefficient and Spearman’s rank correlation analysis.

**Results:**

Within-session intra-rater reliabilities for both examiners 1 and 2 for all vaginal pressures in Tests 1 and 2 were 0.90–0.96 for both perineometers. Between-session intra-rater reliability for the MizCure was 0.72–0.79 for both positions for examiner 1, and 0.63 in the supine position and 0.80 in the standing position for examiner 2. Inter-rater reliability for Test 1 was 0.91 in the supine position and 0.87 in the standing position for the MizCure. The vaginal pressures using the MizCure and Peritron were significantly associated with the supine position (r = 0.68, *P* < .001) and the standing position (r_s_ = 0.82, *P* < .001).

**Conclusion:**

MizCure perineometer is a validated tool to measure PFM strength in both supine and standing positions in healthy nulliparous women.

## Background

An important role of the pelvic floor muscles (PFM) is to maintain urine continence and support the pelvic organs. Voluntary PFM contraction is evaluated by assessing pelvic floor elevation, muscle strength, endurance, and coordination [1]. In clinical practice, digital vaginal palpation is the technique most often used to assess PFM function [2]. In addition, the perineometer and ultrasound imaging are used as the diagnostic tools.

However, the sensitivity of vaginal palpation for quantifying sustained contractions [3] and discriminating variations in force is less than that of other techniques, and it has been shown to have limited reliability even when performed by experienced examiners [4, 5]. Vaginal pressure measurement is a commonly used quantitative evaluation to measure PFM strength [3], and it is significantly lower in women with stress incontinence than in healthy women [6]. It is also used as a teaching tool and as motivation for conducting training exercises [7]. Thus, vaginal pressure measurement has high clinical importance. It is necessary to perform an objective evaluation of the PFM to be able to properly treat, give feedback, and document changes in PFM function during rehabilitation [8]. Additionally, PFM evaluation is recommended by the International Continence Society and considered essential to assess a post-therapeutic intervention effect [9].

The Peritron (Laborie, Mississauga, ON, Canada) perineometer is commonly available for clinical practice and research. However, in order to import the Peritron into some countries, complicated purchasing procedures are required because this device is considered medical equipment. Therefore, there are some barriers to its use for evaluation and research. The MizCure (OWOMED, Seoul, Korea) is sold as a PFM training and biofeedback device. It can be easily purchased online by private individuals. The MizCure is generally used in some urology and gynecology and urogynecology clinics for training. The MizCure uses different units of measurement, which complicates any comparison of measurements obtained between the MizCure and Peritron perineometers. Whether the measurements obtained using the two perineometers, even with probes of similar diameters, are correlated is not known. In a previous study using the Peritron (Cardio-Design, Oakleigh, VIC Australia), it was found to have good inter-rater reliability, intra-rater reliability, and validity [3, 10–12].

However, the reliability and validity of the MizCure have not been verified. The purpose of this study was to clarify the reliability and validity of PFM strength assessment using the MizCure perineometer in healthy women.

## Methods

### Subjects

A convenience sample of 20 healthy women was recruited for this study. A sample size calculation showed that 17 subjects were needed for a correlation greater than 0.7, alpha level of 5%, power of 90%, and effect size of 0.6 [13]. In this study, the sample size was set at 20, taking into account 3 dropouts. This study of intra- and inter-rater reliabilities and agreement was performed at our institute from September 2018 to December 2019. The patients included in this study were nulliparous, non-pregnant women, aged 20–45 years, with body mass index < 25 kg/m^2^ and no gynecological complaints or disease verified, with the ability to correctly contract the PFM. Women with pelvic organ prolapse or who had undergone pelvic reconstructive surgery, those who had symptoms of vaginal infection, intolerance to condoms, or allergy to the gel used in the procedure, as well as those involved in PFM training, were excluded. The present study was approved by the Scientific Ethics Committee of our institute (#018-0056), and all patients provided their informed consent.

### Assessment tools and procedures

#### Assessment tools

##### Manometry 1

**Fig. 1:**
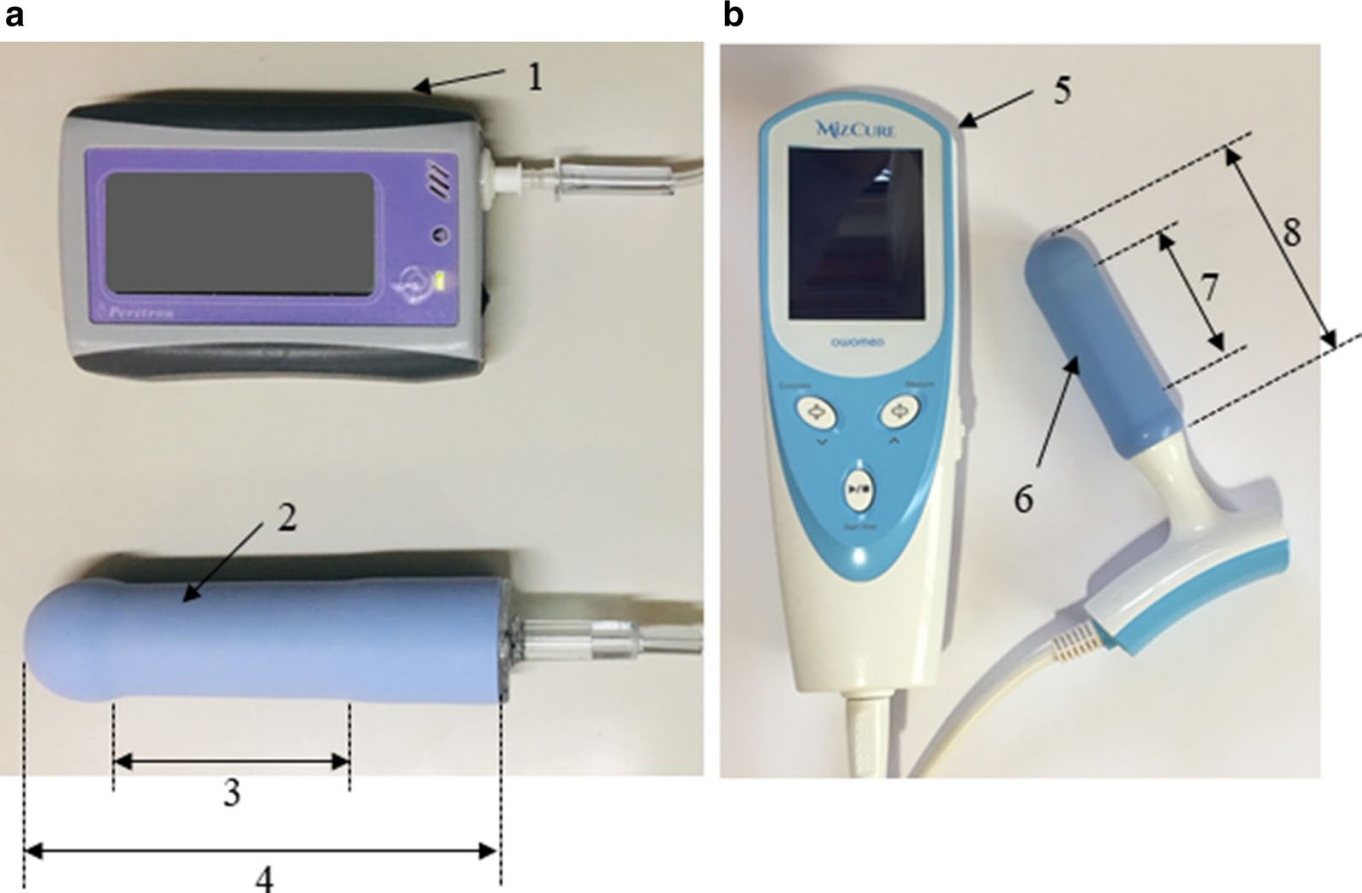
Perineometers used in this study: **a** Peritron 9300 perineometer (Laborie, Mississauga, ON, Canada) with a vaginal probe, 1: Perineometer's main body, 2: Diameter 26–33 (pressurized) mm, 3: Measurable length, 55 mm, 4: Length: 110 mm. **b** MizCure perineometer (OWOMED, Seoul, Korea) with a vaginal probe, 5: Perineometer's main body, 6: Diameter 21–27 (pressurized) mm, 7: Measurable length,50 mm, 8: Length 79 mm. These pictures were taken our devices in our institute. All rights reserved

The Peritron 9300 perineometer (Laborie), shown in Fig. 1, was used in this study. The Peritron perineometer has a conical vaginal probe, 26 mm (pressurized: 33 mm) in diameter and 110 mm in length, with a measurable length of 55 mm. The vaginal probe is connected to the perineometer’s main body with an 80-cm plastic tube. When the probe is compressed by vaginal pressure, a pressure sensor measures the vaginal pressure. The probe consisted of an air-filled silicone rubber sensor and measured pressure in cmH_2_O. The occlusive pressure readings obtained by the perineometer provide surrogate measures of PFM strength.

##### Manometry 2

**Fig. 2:**
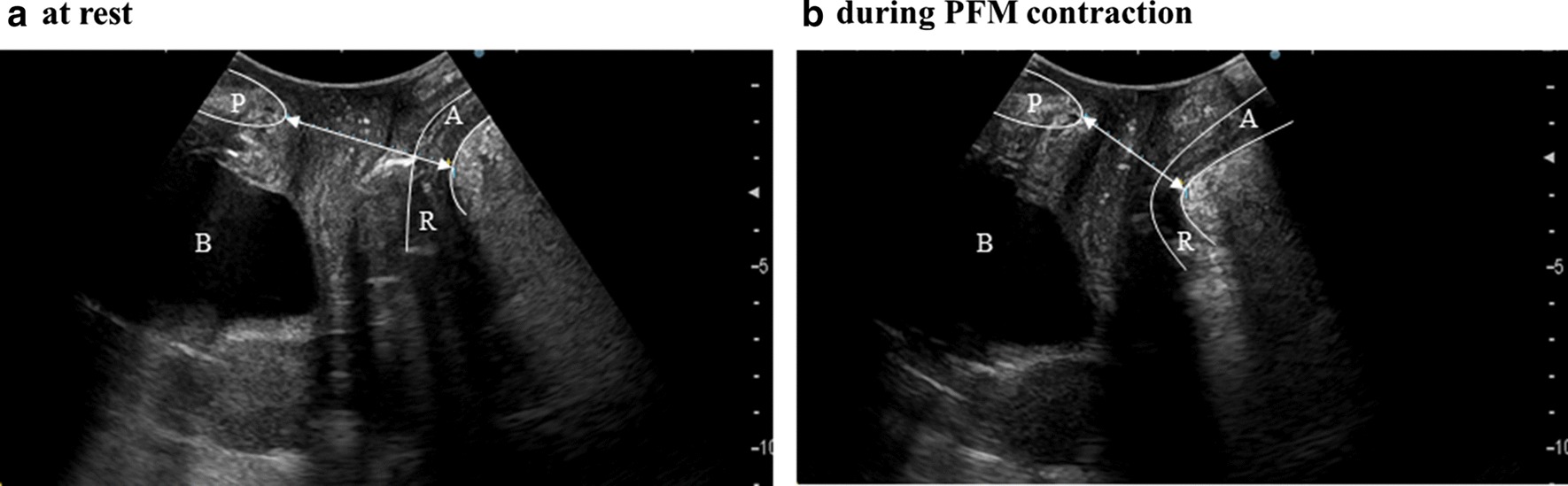
Transperineal mid-sagittal plane ultrasound image in healthy women: At rest (**a**), during pelvic floor muscle contraction (**b**). Urogenital hiatus diameter was measured as the distance between the anorectal junction and the inferior border of the pubic symphysis (white double headed arrow). The levator ani muscle was determined based on the hyperechogenic region posterior to the anorectal junction. The correct pelvic floor muscle contraction indicated the cranial ventral displacement of the levator ani muscle, and urogenital hiatus diameter was shortened. *A* anus, *B* bladder, *P* pubic symphysis, *R* rectum

The MizCure perineometer (OWOMED, Korea) is a conical vaginal insert, 21 mm (pressurized: 27 mm) in diameter and 79 mm in length, with a measurable length of 50 mm (Fig. 2).

The probe was connected to the perineometer’s main body via a 75-cm silicone tube. When a silicone tube is connected to the perineometer’s main body and the power is turned on, air enters the probe and the probe expands. When pressure is applied to the inside of the vagina to the inflated probe sensor, the pressure sensor measures the vaginal pressure. The inflation pressure can be set to 140 or 150 mmHg. In the present study, the inflation pressure was set to 140 mmHg. The unit measured pressure in mmHg.

#### Procedure

##### Test 1

First, before starting the tests, a transperineal technique using two-dimensional (2D) ultrasound confirmed that each woman was able to contract the PFM correctly based on 2D transperineal ultrasound measurements, including the anteroposterior diameter of the urogenital hiatus (measured at rest and during PFM contraction) (Fig. 2). In our group, we demonstrated that 2D transperineal ultrasound is useful to assess PFM function in patients with pelvic organ prolapse [14]. Yang et al. found a close correlation between reduced urogenital hiatus diameter in the sagittal plane and the modified Oxford grading scale [12]. The modified Oxford grading scale is defined as follows: 0 = no contraction; 1 = flicker; 2 = weak; 3 = moderate; 4 = good; and 5 = strong [15]. Second, vaginal pressure was measured with the Peritron and MizCure perineometers. The order of use of the two vaginal manometers and the two test positions were each performed randomly. Testing was conducted with the women in two positions: the supine position, with flexed and slightly abducted legs, and the standing position, with straight and slightly abducted legs. Before data were acquired by the perineometers, the participant inserted the probe, which was covered with a condom and lubricated with hypoallergenic gel, into her vaginal cavity. The participants were instructed to place the probe inside the vagina to a location where 0.5–1.0 cm of the probe was visible from the outside of the introitus. PFM strength was then evaluated by a maximum voluntary contraction, as measured by squeeze pressure. The instruction used for each contraction was ‘squeeze and lift the PFM as much as you can’. Vaginal pressure testing was performed with three repetitions of maximum voluntary contractions that each lasted for 3 s, with a 3-s rest between contractions. A 2-min rest break was then taken. Visible co-contraction of the transversus abdominis muscle was permitted, as long as there was no pelvic tilting [16, 17]. Examiner 1 was a physiotherapist with 11 years of clinical experience. Examiner 2 was a physiotherapist with 5 years of clinical experience and 4 years of educational experience in a university institution.

##### Test 2

All women were evaluated twice. After the testing protocol was completed in the first session (Test 1), all subjects repeated the protocol 2–6 weeks later (Test 2). In Test 2, vaginal pressure measurements were performed using only the MizCure perineometer. The order of the two test positions (supine, standing) and the two examiners were each assigned randomly.

### Statistical analysis

Within- and between-session intra-rater reliabilities in vaginal pressure values (maximal voluntary contraction) were analyzed using intraclass correlation coefficients (ICC) (1, 1), and inter-rater reliability was analyzed using ICC (2, 1). Validity was assessed by Pearson’s product-moment correlation coefficient and Spearman’s rank correlation analysis of the vaginal pressure values of the Peritron and MizCure. Pearson's product-moment correlation coefficient was used when the data were normative, and Spearman's rank correlation coefficient was used when the data were non-normative. Statistical analyses were performed using the free statistical analysis software R, version 2.12.0 (https://personal.hs.hirosaki-u.ac.jp/pteiki/research/stat/S/), with the level of significance set at 5%.

## Results

The median age of the 20 healthy female participants was 26.5 years (range 23–45 years), and their median body mass index was 19.4 (range 17.5–23.4) kg/m^2^. None of the subjects included in the analysis had done PFM training before participating in the research project or between the two evaluation points. Table 1 summarizes within and between-session vaginal pressure values obtained by examiners 1 and 2 (MizCure and Peritron perineometers). All raw data are referred to as Additional file 1.

**Table 1:** Mean and standard deviation of vaginal pressure values and within-session intra-rater reliability analysis for vaginal pressure values for examiners 1 and 2 (N = 20)

	Supine position		Standing position	
	Mean ± SD	ICC (1, 1) (95% CI)	Mean ± SD	ICC (1, 1) (95% CI)
Examiner 1				
Test 1				
Peritron (cmH_2_O)	37.5 ± 14.6	0.96 (0.93–0.98)	36.7 ± 10.3	0.95 (0.90–0.97)
MizCure (mmHg)	25.1 ± 8.1	0.92 (0.85–0.96)	25.0 ± 9.0	0.92 (0.84–0.96)
Test 2				
MizCure (mmHg)	25.1 ± 8.1	0.91 (0.82–0.96)	25.0 ± 9.0	0.93 (0.87–0.97)
Examiner 2				
Test 1				
Peritron (cmH_2_O)	36.4 ± 14.8	0.94 (0.89–0.97)	37.7 ± 10.5	0.94 (0.89–0.97)
MizCure (mmHg)	26.5 ± 8.3	0.90 (0.80–0.95)	24.6 ± 10.7	0.95 (0.91–0.98)
Test 2				
MizCure (mmHg)	25.5 ± 8.4	0.90 (0.82–0.95)	27.2 ± 10.6	0.93 (0.87–0.97)

Values are presented as mean ± standard deviation (SD)

Test 2 was performed 2–6 weeks after Test 1

*ICC* intraclass correlation coefficient, *CI* confidence interval

### Within-session intra-rater reliability

Table 1 shows the within-session intra-rater reliability using three repetitions of each maximum voluntary contraction by the MizCure and Peritron perineometers for both Tests 1 and 2. For both examiners 1 and 2, all vaginal pressures in Tests 1 and 2 had ICC (1, 1) values of 0.90–0.96.

### Between-session intra-rater reliability

Between-session intra-rater reliability values for the MizCure perineometer for examiners 1 and 2 are shown in Table 2. The between-session intra-rater reliability of examiner 1 was ICC (1, 1) = 0.72 for the supine position and 0.79 for the standing position. The between-session intra-rater reliability of examiner 2 was ICC (1, 1) = 0.63 for the supine position and 0.80 for the standing position.

**Table 2:** Between-session intra-rater reliability analysis for the MizCure perineometer for examiners 1 and 2

	ICC (1, 1) (95% CI)	
	Supine position	Standing position
Examiner 1	0.72 (0.43–0.88)	0.79 (0.55–0.91)
Examiner 2	0.63 (0.28–0.83)	0.80 (0.57–0.91)

*ICC* intraclass correlation coefficient, *CI* confidence interval

### Within- and between-session inter-rater reliabilities

Table 3 shows the inter-rater reliability analysis for vaginal pressure values for Tests 1 and 2. The inter-rater reliability for Test 1 was ICC (2, 1) = 0.96 for both the supine and standing positions for the Peritron. The ICC (2, 1) for MizCure was 0.91 for the supine position and 0.87 for the standing position. The inter-rater reliability of the MizCure in Test 2 was ICC (2, 1) = 0.69 for the supine position and 0.95 for the standing position.

**Table 3:** Inter-rater reliability analysis for vaginal pressure values for tests 1 and 2

	ICC (2, 1) (95% CI)	
	Supine position	Standing position
Test 1		
Peritron	0.96 (0.91–0.98)	0.96 (0.89–0.98)
MizCure	0.91 (0.79–0.96)	0.87 (0.72–0.95)
Test 2		
MizCure	0.69 (0.38–0.86)	0.95 (0.89–0.98)

*ICC* intraclass correlation coefficient, *CI* confidence interval

### Validity

Significant correlations between the Peritron and MizCure perineometers in the measurements of vaginal pressure were found in the supine position (Pearson’s correlation coefficient of 0.68, *P* < 0.001) and in the standing position (Spearman’s correlation coefficient of 0.82, *P* < 0.001). More details about these results are presented in Table 4.

**Table 4:** Correlation analysis of measurements in the supine position and the standing position

	Correlation coefficient	*P* value
	r or r_s_	
Supine position	0.68^a^	< .001
Standing position	0.82^b^	< .001

^a^Pearson’s product-moment correlation coefficient for MizCure vs Peritron

^b^Spearman’s rank correlation coefficient for MizCure vs Peritron

## Discussion

PFM training has been shown to improve stress urinary incontinence and pelvic organ prolapse [18, 19] and is recommended by the International Continence Society as Grade A [20]. However, about 30% of women report failure to contract the PFM correctly [21]. Incorrect PFM contraction is not expected to have a training effect. Therefore, it is recommended that proper PFM training should always include an objective assessment of correct contraction.

In the present study, the reliability and validity of PFM strength assessment using the MizCure perineometer were examined in healthy women. Using transvaginal devices, it is known that the measurement of PFM strength depends on the size and placement of the probe, the subject’s cooperation, and the examiner’s experience and skills [22, 23]. If a small perineometer probe is used, the placement of the intravaginal probe causes reliability problems, because the probe may be located not completely adjacent to the pressure zone [24]. Thus, in the current study, whether the MizCure can properly measure PFM strength was verified using the widely reported Peritron.

In the previous study on the intra-rater reliability of the Peritron, the ICC (1, 1) was over 0.9 for the supine and standing position measurements [3]. Therefore, the results of the present study suggest that the within-session intra-rater reliability of the MizCure is as good as that of the Peritron. Rahmani et al. and Ferreira et al. reported the inter-rater reliability of vaginal pressure measurements using the Peritron. Rahmani et al. reported that the between-session ICC (1, 1) was 0.88 [10]. Ferreira et al. reported that the mean vaginal squeeze pressure had good inter-rater reliability, and the difference between the examiners was not significant [11]. In the current study, the between-session intra-rater reliability of the MizCure was slightly lower than that reported by Rahmani et al. [10]. This is primarily because of the size and placement of the probes. In the current study, the insertion and placement of the probe were performed by the subject. The MizCure has a smaller probe than the Peritron, and it is difficult to standardize the position of the probe between subjects. The previous study showed that probe placement can affect measurement results [22]. The fact that intravaginal squeeze pressure changes along the vagina [25] means that the profile of vaginal pressure is not fully understood. This suggests that probe placement may have affected reliability. The MizCure probe used in the current study was 79 mm in length. The vaginal high-pressure zone at rest and during PFM contraction is 2–4 cm from the vaginal introitus [26]. The vaginal high-pressure zone was shown to be related to the PFM contraction [25]. Therefore, the MizCure probe is considered to cover the high-pressure zone of the vagina. The other factor that may have affected the results for the reliability of vaginal pressure measurements may be the skill of the examiner. With respect to this factor, the two examiners were skilled physical therapists with urologic education. The examiner checked the correct PFM contraction with 2D ultrasound prior to measurement, and always checked to prevent compensatory movement due to PFM contraction (e.g., pelvic tilt or excessive abdominal muscle contractions). Therefore, the effect of the two examiners on the measured value was thought to be small.

ICC reliability criteria are defined as substantial for 0.61–0.80 and almost perfect for 0.81–1.0 [27]. From this perspective, the present results indicate that the intra-rater reliability of the MizCure within and between days was substantial to almost perfect. The inter-rater reliability was similar. These results suggest that the use of the MizCure perineometer to evaluate PFM strength can minimize the effects of the examiner and provide good intra-rater reliability.

The validity of the MizCure was evaluated using Pearson’s product-moment correlation coefficient and Spearman’s rank correlation coefficient. The correlation between MizCure and Peritron measurements has not been previously reported. The MizCure measurements showed a significant correlation with the Peritron measurements: r = 0.68 for the supine measurements and r_s_ = 0.82 for the standing measurements. For the correlation coefficient, 0.4–0.69 is considered moderate and 0.7–0.89 high [28]. Therefore, the correlation coefficients for the MizCure were moderate to high. The results suggest that the MizCure is a suitable tool for measuring PFM strength. There have been reports in the past examining the validity of the Peritron and other perineometers [29]. Barbosa et al. reported the following differences among the other devices: acquisition of higher or lower measurement values depends not only on the diameter of the probes used, but also on other variables, such as individual vaginal diameter, probes of different materials, and differences in the sensitivity of each piece of equipment to vaginal pressure [29]. Therefore, a detailed examination of the characteristics of the MizCure perineometer and the individual’s vagina will be necessary in the future.

A recent paper in the statistical analysis of correlation coefficients for reliability suggests different cut-off values for ICC. Based on the 95% confidence interval of the ICC estimate, values less than 0.5, values between 0.5 and 0.75, values between 0.75 and 0.9, and values greater than 0.90 indicate poor, moderate, good, and excellent reliability, respectively [30]. Applying the results of this study, the 95% confidence intervals for inter-rater reliability for supine position measurement were 0.43–0.88 for examiner 1 and 0.28–0.83 for examiner 2, as shown in Table 2. In addition, as shown in Table 3, the 95% confidence interval for inter-rater reliability in the supine measurement was 0.38–0.86. This shows that the lower limit of the 95% confidence interval is low, and the variability is high. This is the lower bound of the 95% confidence interval is low. Due to the small number of subjects in this study, this indicates a large variation in the confidence interval. In the future, it is necessary to examine the results with a larger population.

The advantage of the MizCure perineometer is that it is easy to purchase, portable, and simple to use. These results suggest that the MizCure is a tool that can quantitatively reflect PFM function, since the reliability and validity of the measured vaginal pressure values are good. This may help select the measurement position for evaluation and treatment purposes and help the treatment plan. From the current study, the MizCure might be a device that can be used by physical therapists, nurses, and physicians involved in pelvic floor rehabilitation to assess PFM function.

However, as a limitation of this study, the results of the present study are limited to healthy nulliparous women with normal BMI without PFM dysfunction, such as stress urinary incontinence or pelvic organ prolapse. And all subjects were not asked about their sexual activity prior to vaginal pressure measurement. This may affect the results of vaginal pressure measurements. An additional limitation is the small number of subjects. A small sample size reduces the power of the study and increase the margin of error. Future study must be needed to ensure our study.

## Conclusion

The present findings suggest that MizCure perineometer is a validated tool to measure PFM strength in both supine and standing positions in healthy nulliparous women.

## Supplementary information

**Additional file 1.** All raw data.

## Data Availability

The datasets used and/or analyzed during the current study are available from the corresponding author on reasonable request.

## Abbreviations

PFM: Pelvic floor muscles
2D: Two-dimensional
ICC: Intraclass correlation coefficient
